# Paternal Smoking and Risk of Childhood Acute Lymphoblastic Leukemia: Systematic Review and Meta-Analysis

**DOI:** 10.1155/2011/854584

**Published:** 2011-05-29

**Authors:** Ruiling Liu, Luoping Zhang, Cliona M. McHale, S. Katharine Hammond

**Affiliations:** Division of Environmental Health Sciences, School of Public Health, University of California, Berkeley, CA 94720, USA

## Abstract

*Objective*. To investigate the association between paternal smoking and childhood acute lymphoblastic leukemia (ALL). *Method*. We identified 18 published epidemiologic studies that reported data on both paternal smoking and childhood ALL risk. We performed a meta-analysis and analyzed dose-response relationships on ALL risk for smoking during preconception, during pregnancy, after birth, and ever smoking. *Results*. The summary odds ratio (OR) of childhood ALL associated with paternal smoking was 1.11 (95% Confidence Interval (CI): 1.05–1.18, *I* ^2^ = 18%) during any time period, 1.25 (95% CI: 1.08–1.46, *I* ^2^ = 53%) preconception; 1.24 (95% CI: 1.07–1.43, *I* ^2^ = 54%) during pregnancy, and 1.24 (95% CI: 0.96–1.60, *I* ^2^ = 64%) after birth, with a dose-response relationship between childhood ALL and paternal smoking preconception or after birth. *Conclusion*. The evidence supports a positive association between childhood ALL and paternal ever smoking and at each exposure time period examined. Future epidemiologic studies should assess paternal smoking during well-defined exposure windows and should include biomarkers to assess smoking exposure and toxicological mechanisms.

## 1. Introduction

Leukemia is the most common cancer in children and adolescents, accounting for about 1 out of 3 cancers in children [1]. Each year, around 3,250 children are diagnosed with leukemia, of which about 2,400 are acute lymphoblastic leukemia (ALL) cases [2]. In the USA, survival rate for children with ALL has improved markedly since the early 1970s and is now approximately 80%, but incidence rates have not decreased and have, in fact, increased by 0.8% annually from 1975 to 2007 [3]. Worldwide, according to the World Health Organization (WHO), there were 33,142 deaths from leukemia among children under age 15 in 2004, and childhood (<15 years) leukemia caused 1,228,075 disability adjusted life years (DALYs) [4]. Identifying risk factors for childhood leukemia is an important step in the reduction of the overall burden of childhood diseases.

Though it has been studied intensively, the etiology of childhood leukemia is not well established. A two-hit model was proposed by Greaves in which prenatal chromosome translocations and postnatal genetic alterations are necessary for childhood leukemia development [5]. Genetic susceptibility and environmental factors play potential roles in this process [6]. Ionizing radiation has been significantly linked to childhood leukemia [7]; evidence for an association with benzene or with parental smoking and alcohol consumption is less convincing. 

Multiple studies on parental smoking and childhood leukemia have been conducted in the past two decades, probably because tobacco smoke is a well-documented and prevalent carcinogen. Despite ongoing global efforts to reduce tobacco use, one billion men and 250 million women currently smoke worldwide [8], causing 5 million deaths and 57 million DALYs from cancer and other diseases each year [9]. In the USA, 46 million people or 24% of all adults smoke [10], which caused nearly half a million deaths and 5 million years of potential life lost each year from 2000 to 2004 [11]. In China, though smoking is uncommon among women, almost two thirds of men smoke [12], causing one million deaths each year to smokers [13] and 56,000 deaths and 480,000 DALYs from lung cancer and ischemic heart disease to nonsmokers [14]. 

At least 250 chemicals in tobacco smoke are known to be toxic or carcinogenic, including volatile organic chemicals like benzene, formaldehyde, aromatic amines, polycyclic aromatic hydrocarbons (PAHs), and nitrosamines and radioactive compounds like polonium 210 [15]. Benzene has been shown to affect the blood-forming system at low levels [16], and formaldehyde has been shown to increase leukemia risk among exposed adults [17]. Smoking is causatively linked with adult leukemia [18], and secondhand smoke (SHS) is qualitatively similar in its chemical constituents to mainstream smoke [15], indicating that SHS exposure has the potential to cause adverse effect on the hematopoietic system. Children aged 6 to 11 years were reported to have urinary concentrations of the tobacco-specific carcinogen nitrosamine 4-(methylnitrosamino)-1-(3-pyridyl)-1-butanol (NNAL) nearly four times those of adult nonsmokers [19], indicating that children are less able to avoid exposure to SHS than adults. Smoking has also been shown to affect sperm morphology, motility, and concentration and to increase oxidative damage to sperm DNA [20]. Together, these findings indicate that parental smoking is a potential risk factor for childhood leukemia that could induce DNA damage and mutation pre- and postnatally. However, epidemiological studies on this topic have reported inconsistent findings. Through 2009, 20 studies [21–40] investigated maternal smoking and childhood ALL, with three studies [27, 36, 39] reporting statistically positive associations, two [32, 40] reporting statistically negative associations, and the remainder reporting nonsignificant association. Among the 18 studies on paternal smoking, eight showed increased risks of childhood ALL for at least one index of paternal smoking [23, 27, 33, 34, 36, 37, 41, 42]. 

Given the extent of the exposure, the known carcinogenicity of tobacco smoke, and the inconsistent findings for paternal smoking and childhood leukemia risk, a thorough examination of the causal association between paternal smoking and childhood ALL, a major type of leukemia in children, is necessary. A recent meta-analysis by Lee et al. [42] found a significantly positive but weak association between paternal smoking preconception (but not after birth) and risk of childhood leukemia and ALL [42]. This analysis was based on 11 studies published from 1990 to 2008, plus their own study, results of which were published concurrently with the meta-analysis. However, Lee's meta-analysis did not include all published studies and did not give enough details of study exclusion criteria. Also, they did not look at confounding adjustments or perform assessment of dose-response relationships. Further, a new study [33] was published after this meta-analysis had been accepted for publication. Here, we conduct an updated and more comprehensive meta-analysis of the association between paternal smoking and childhood ALL based on 18 published studies that reported risk estimates or that provided data to calculate risk estimates. We examined risks associated with paternal smoking preconception, during pregnancy and after birth and, for the first time, analyzed dose-response relationships of exposure in these time windows.

## 2. Methods and Analysis

### 2.1. Selection of Studies

Preliminary literature searches were conducted by searching for the topics “smok*” or “tobacco” or “cigarette” and “leukemia” and “child*” in the ISI Web of Knowledge and PubMed databases. After duplicates were identified and removed, the titles and abstracts of the remaining records were examined, and all reviews and original epidemiologic studies investigating risk factors of childhood leukemia were included for further examination on the availability of information on paternal smoking and leukemia. The bibliographies or citations of all relevant articles were also searched and cross-referenced. Original epidemiologic studies published in peer-reviewed scientific journals or edited books, with data available on both childhood ALL and paternal smoking, were included. 

To be included in this meta-analysis, studies had to fulfill three criteria: (1) reported estimates of association (odds ratio, OR, or relative risk, RR) of paternal smoking, and ALL, (2) reported estimated variance (e.g., 95% confidence intervals, 95% CI hereafter) or included data needed to calculate it, and (3) did not present data from the same group of subjects as another publication used in the meta-analysis (in which case, the article with the most appropriate exposure assessment or published most recently was selected).

### 2.2. Data Analysis

#### 2.2.1. Definition of Exposure Time Windows

Based on data available in the studies included in the analysis, three exposure time windows of paternal smoking with potential relevance to the development of ALL were defined, that is, *preconception*, *during pregnancy* and *after birth*. Summary effects were estimated for each of these three exposure windows. To estimate the overall summary effect of paternal smoking in any time window, the risk estimate was selected from each study included in the following order of preference: paternal ever smoking in lifetime, paternal smoking before conception, during pregnancy, and after birth. For the four studies which used exposure time windows covering more than one of these windows, the same estimate of risk was used for all the narrower time windows falling into the wider time window. For example, if a study presented only the relative risk estimate for paternal smoking in the year or 12 months prior to birth, that value was used to estimate the summary effect of both paternal smoking before and during pregnancy. In one study by Brondum et al. [21], the time window of pregnancy was further divided into three trimesters, and data were available only for each trimester rather than the whole time window. Since data were not available to combine the effects for all the three trimesters, and the relative risk reported for each trimester was almost the same, data from the first trimester was selected to represent this time window.

#### 2.2.2. Selection of Outcomes and Exposure Indices

Most studies provided data on ALL specifically. In one study which reported the risk of three immunological subtypes of ALL, common-ALL, pre-B-ALL, and T-ALL, but not the risk of ALL overall, and no data were available to estimate the overall relative risk of ALL [43], the estimate for the most common subtype (common-ALL, which comprised 66% of the ALL cases in the study) was selected. 

When both multiple and binary exposure categories were available, the category with the highest exposure was selected to estimate the summary effect. Although multiple exposure indices were used in published studies, the majority of studies used the exposure index of cigarettes per day, thus the exposure index chosen from each study for this analysis was in the following order of preference: cigarettes per day (CPD), pack year (PY), number of smoking years, and smoker/nonsmoker. When both continuous and categorical exposure indices were available, categorical indices were selected for the point estimate and the continuous measures were used for dose-response analysis. When both current and ever smoking status were available, the current smoking status was selected.

#### 2.2.3. Calculation of Summary Effects

Both fixed-effect and random-effect models were used to calculate summary effects. The fixed-effect model uses the inverse variance weighting method [44], and the variance (95% CI) of the summary effect estimate was calculated using the method presented by Shore et al. [45] if the estimate on the confidence interval was wider than the one estimated by the fixed-effect model itself. The Shore correction incorporates between-study heterogeneity and is usually more conservative than the fixed-effect model in estimating the variance. The random-effect model allows for the incorporation of between-study heterogeneity (if it is present) into the summary variance estimate (95% CI) [46]. Results from random-effect models were used for interpretations when between-study heterogeneity was statistically significant, otherwise results from fixed-effect models with Shore-corrected 95% CIs were used when the CIs were wider than the uncorrected ones estimated by fixed-effect models.

#### 2.2.4. Subgroup Analysis

Analysis was also conducted to estimate summary effects for different study subgroups, such as those with the highest index of exposure categories, with adjusted risk estimates, with well-defined exposure time windows or with population based controls, in order to investigate the sensitivity of estimated summary effects to factors defining the subgroups.

#### 2.2.5. Dose Response Analysis

All studies with dose-response data were included for review and analysis. ORs for paternal smoking of ≥20 CPD during each time window from different studies were extracted, or estimated if raw data were available, and combined to get a summary estimate of OR for paternal smoking of ≥20 CPD in this time window. Similarly, summary estimates of OR for paternal smoking of 10–19 CPD or <10 CPD (<20 CPD for the time window of after child birth) were calculated, and these summary ORs were plotted and compared for each time window.

#### 2.2.6. Heterogeneity Analysis

Heterogeneity among studies was assessed using the general variance-based method as described by Petitti [47] and using the *I* ^2^ [48], which describes the percentage of total variation across studies that is due to heterogeneity rather than chance, and which is calculated as *I* ^2^ = 100% × (*Q* − *df*)/*Q*, where *Q* is Cochran's heterogeneity statistic and *df* the degrees of freedom, with negative values of *I* ^2^ set to zero. The Cochran's heterogeneity statistic is known to have low power of detecting true heterogeneity when the number of studies is small, while *I* ^2^ does not inherently depend on the number of studies in the meta-analysis [48]. Low, moderate, or high degree of heterogeneity was suggested to be approximated by *I* ^2^ values of 25%, 50%, and 75%, respectively [48].

#### 2.2.7. Analysis of Publication Bias

Publication bias arises when studies with statistically significant positive results for exposure to environmental pollutants are more likely to be published and cited [49]. In this meta-analysis, publication bias was assessed by using funnel plots and Egger's and Begg's tests [50, 51] and by estimating the proportion of papers which reported statistically nonsignificant risk assessments.

Funnel plots (plots of effect estimates against sample size) are usually skewed and asymmetrical in the presence of publication bias and other biases [52]; Egger's test [51] is a linear regression approach to measure funnel plot asymmetry. Begg's test [50] assesses the interdependence of variance and effect size using Kendall's rank correlation test. This bias indicator makes fewer assumptions than that of Egger's test, but it is not sensitive to as many types of bias as Egger's test. If the number of studies included for Egger's or Begg's tests is small, the power of detecting publication bias could be very low [50, 51].

All data analyses described above were conducted using StataIC11.

## 3. Results

### 3.1. Description of Studies Included for This Updated Meta-Analysis

Twenty-one original epidemiology studies that examined risk factors of childhood leukemia and reported data on both ALL and paternal smoking were found. Three studies did not report relative risk of ALL for any index of paternal smoking or raw data to calculate the risk [53–55], and they were excluded for this study. Therefore, a total of 18 studies were included in the final analysis, and these studies are summarized in Table 1. The studies were conducted in 8 different countries, and their results were all published in peer-reviewed journals from 1990 to 2009. All studies were case-control studies, probably because childhood leukemia is too rare to conduct a cohort study. The age of childhood leukemia patient inclusion varies as detailed in Table 1, with studies including cases through age 18 months (*n* = 1), through age 9 years (*n* = 1), through age 14 years (*n* = 10), through age 15 years (*n* = 4), through age 18 years (*n* = 1), or unspecified with mean age 6.1 years and standard deviation 3.6 years for cases and mean age 6.6 years and standard deviation 3.5 years for controls (*n* = 1). Controls were recruited from the general population in all but three studies which used hospital-based controls [29, 31, 42]. All studies except the one by Magnani et al. 1990 [29] matched controls and cases by age and most studies also matched by gender and area of residency. Exposure information on paternal smoking was obtained primarily by interviewing the mother (11 studies [23, 29–36, 38, 42]), while the remaining studies interviewed both parents, when possible.

Three studies reported childhood ALL risks in relation to parental use of tobacco [35, 36, 38] using data from the same large project called the Oxford Survey of Childhood Cancers (OSCC). This survey interviewed the parents of all children who died of cancer (including leukemia) before their sixteenth birthday in England, Wales, and Scotland during the period 1953 to 1984 and parents of population-based healthy control children, matched for sex and date of birth [35, 36, 38]. Because each of the three papers reported results from different and non-overlapping subsets of data, they were regarded as independent and were all included in the meta-analysis. There was, however, a small degree of overlap between cases included by Sorahan et al. 1995 [35] from the OSCC and cases included by Sorahan et al. 2001 [37]. The later publication included 139 *newly diagnosed* childhood ALL cases less than 15 years old in three areas in England from 1980 to 1983 [37], and the early paper included 371 children who *died* from ALL before their sixteenth birthday in England, Wales, and Scotland between 1977 to 1981 [35]. Thus, there is potential overlap between newly diagnosed ALL cases and those who died from ALL during 1980-1981. Given the high five-year survival rate of childhood ALL during that time period in England (about 50%) [56], such an overlap would be expected to be very small in this 2 year period, thus, both the Sorahan et al. studies [35, 37] were included in this analysis. 

Of the 18 studies included in the analysis, 6 reported data on the risk of childhood ALL associated with paternal ever-smoking throughout the lifetime [21, 23, 28, 36, 38, 42]. The summary effects of paternal smoking preconception, during pregnancy and after the child birth could be estimated from 13 studies, 8 studies and 7 studies, respectively. Menegaux et al. 2005 [31] reported that paternal smoking was not associated with ALL either before or during pregnancy, but did not provide the actual data. However, they did report data on the association during the period from the child birth to the interview. Thus, the Menegaux study (2005) was included to calculate the summary effects of exposure after birth only.

### 3.2. Estimates of Summary Effects, Subgroup Analysis and Heterogeneity Analysis

#### 3.2.1. Overall and Lifetime Paternal Smoking

**Figure 1:**
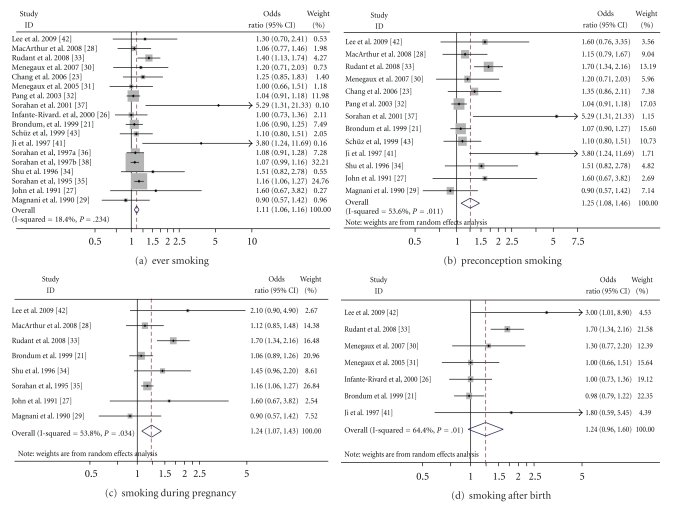
Meta-analysis of the association between childhood ALL and paternal smoking in different time windows. Random-effect OR estimates and weights were used in the graphs. *X*-axis represent the OR (odds ratio). The sizes of the boxes indicate the weight of the corresponding study used for estimates of summary effects.

Results of the meta-analysis are presented in Figure 1 and Table 2. Figure 1 graphs the ORs (random effects analysis) generated by each meta-analysis and the ORs and weights of the individual studies included therein. Table 2 details the summary relative effects of paternal smoking overall and during specific time windows, and for different subgroups within these exposure windows, using both fixed effect and random effect models. The degree of heterogeneity associated with each measure is also provided. The summary effect for paternal ever smoking at any time period was 1.11 (95% CI: 1.05–1.18, *I* ^2^ = 18%) based upon all 18 studies, shown in Figure 1(a) and Table 2. When analysis was restricted to the data from six studies on overall lifetime paternal smoking status, only, not during specific exposure windows, the summary effect decreased from 1.11 to 1.07 (95% CI: 1.01–1.14, *I* ^2^ = 0%).

#### 3.2.2. Preconception Paternal Smoking

The summary OR for risk of ALL associated with preconception smoking was 1.25 (95% CI: 1.08–1.46, *I* ^2^ = 53%) based on 13 studies (Figure 1(b) and Table 2). When only the highest exposure indices available in 10 studies were included, the summary effect increased to 1.38 (95% CI: 1.11–1.72, *I* ^2^ = 45%, Table 2). Exclusion from the analysis of studies with the largest or smallest OR, those with the highest weight, or those with hospital-based controls, did not have a large impact on either the summary effect estimates or the heterogeneity. Both the summary effect and the heterogeneity between studies decreased (1.17, 95% CI: 1.02–1.35, *I* ^2^ = 33%) after removing studies with exposure time windows that spanned more than those defined in our analysis (with wide defined exposure time windows hereafter, for example, the year prior to the child's birth to the time of interview [33], 12 months prior to the child birth [27], or up to child's birth [29]). Exclusion of Rudant et al. 2008 [33] alone, whose estimated effect (OR = 1.7; 95% CI: 1.3–2.1) was for the highest paternal smoking ≥20 cigarettes/day from the year prior to the child birth to the time of interview, had a similar summary effect on OR and heterogeneity as removing all 3 studies with wide defined exposure windows. Five studies reported the effect of paternal smoking specifically in the last one or three months before pregnancy [21, 23, 30, 34, 43], and their summary OR was 1.13 (95% CI: 0.98–1.29) with no evidence of heterogeneity between studies (*I* ^2^ = 0%).

#### 3.2.3. Paternal Smoking during Pregnancy

Children whose fathers smoked while they were *in utero* had a 24% higher relative risk of getting ALL than those whose father did not (summary OR = 1.24, 95% CI: 1.07–1.43, *I* ^2^ = 54%), shown in Figure 1(c) and Table 2. When only the highest exposure indices available in four studies were included, the summary effect was 1.28 (95% CI: 0.93–1.76, *I* ^2^ = 65%). Inclusion of only studies with adjusted ORs also increased the summary effect (summary OR = 1.34, 95% CI: 1.07–1.68, *I* ^2^ = 60%). In contrast, when only studies with well-defined exposure time windows (during pregnancy) were included, the summary effect decreased to 1.15 (95% CI: 1.06–1.23, *I* ^2^ = 0%). Exclusion of Rudant et al. 2008 [33] alone, which estimated the effect (OR = 1.7; 95% CI: 1.3–2.1) for the highest paternal smoking of ≥20 CPD from the year prior to the child birth to the time of interview, had a similar effect on OR and heterogeneity as removing all 3 studies with widely defined exposure window. Removing extreme ORs, studies with the highest weight or with hospital-based controls had little effect on the summary estimates (Table 2).

#### 3.2.4. Paternal Smoking after Birth

Paternal smoking after birth also had a positive but borderline significant association with childhood ALL (OR = 1.24; 95% CI: 0.96–1.60, *I* ^2^ = 64%), shown in Figure 1(d) and Table 2. Little difference was observed in the summary effects when removing extreme ORs or hospital-based studies (Table 2). When only studies with the highest exposure indices were included, or the study with the highest weight was excluded, the summary OR increased to 1.33 (95% CI: 1.00–1.78, *I* ^2^ = 57%). As with the other exposure windows, exclusion of Rudant et al. 2008 alone, reduced the OR, in this case, to 1.06 (95% CI: 0.89–1.26) and the heterogeneity to 12%.

### 3.3. Effect of Adjustment for Confounding Factors

One study [38] reported unadjusted RR, and two studies [35, 36] did not clarify whether they had adjusted for any other variables in their effect estimates. All remaining studies adjusted for at least some index of social economic status, for example, income or parental education, and most studies also adjusted for other potential confounders including residential area, birth weight, parental age and/or race/ethnicity, and alcohol drinking during pregnancy. For preconception paternal smoking, eight studies [23, 27, 28, 30, 33, 34, 37, 42] presented numbers of cases and controls with or without exposure allowing for the calculation of crude ORs. The current meta-analysis on these eight studies showed that the summary OR of the calculated crude ORs was 1.46 (95% CI: 1.18–1.80) and the summary OR of the reported ORs with adjustment was 1.37 (95% CI: 1.09–1.71).

### 3.4. Analysis of Publication Bias

**Figure 2:**
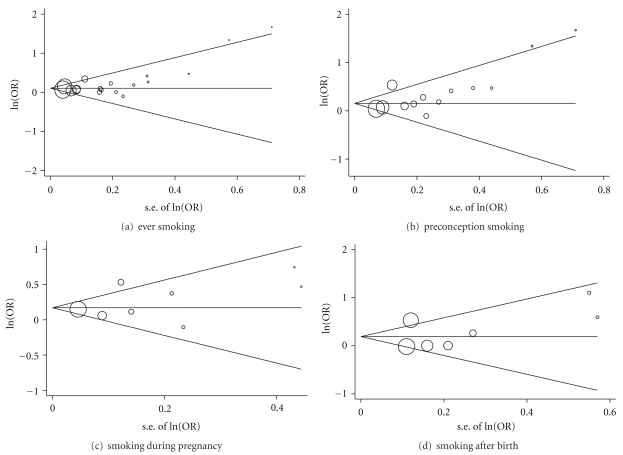
Begg's funnel plots of the log odds ratio (ln(OR)) versus the standard error of the log odds ratio (s.e of ln(OR)) of the studies used in the meta-analysis. Random-effect model OR estimates were used in the graphs. The sizes of the circles indicate the inverse-variance weight of the corresponding study.

For preconception paternal smoking, the *P* values for Begg's and Egger's tests were 0.035 and 0.007, respectively, which, together with the funnel plots (Figure 2(b)), suggest some evidence of publication bias. This might be due to the inclusion of two relatively smaller studies with greater ORs and variance of estimates [37, 41]. Ji et al. 1997 [41] reported an OR of 3.8 (95% CI: 1.3–12.3) for children whose fathers smoked for 5 pack years before conception; Sorahan et al. 2001 [37] reported an OR of 5.29 (95% CI: 1.31–21.3) for paternal smoking with ≥40 CPD before pregnancy. Removal of these two studies resulted in a *P* value of 0.24 for Begg's test and 0.07 for Egger's test, while the summary effect did not change much (random-effect model, summary OR = 1.20, 95% CI: 1.05–1.36, *P* = .007, *I* ^2^ = 41%). Similar publication bias test results were found for paternal ever smoking in any time period, most likely because the same estimates from the Ji et al. 1997 [41] and Sorahan et al. 2001 [37] were included, Figure 2(a). 

Both Begg's and Egger's tests showed no evidence of publication bias for studies on paternal smoking during pregnancy and after child birth (both with *P*  value > .1), though the power to detect publication bias might be lower because of the smaller number of studies included compared with preconception exposures, Figures 2(c) and 2(d). Nevertheless, the fact that only eight of the 18 studies included reported any statistically significant effect of paternal smoking on childhood ALL risk further indicates that the probability of publication bias is small.

### 3.5. Dose Response Analysis

**Figure 3:**
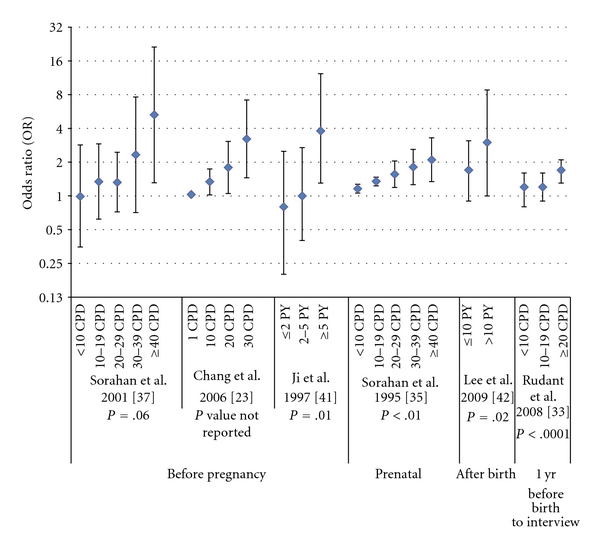
Clear positive dose-response associations between paternal smoking and childhood ALL found from the literature. Note: Among all the 18 studies included in this meta-analysis, two did not present dose-response analysis [32, 43], 10 did not find clear dose-response trend, and the remaining six studies reported clear dose-response trends [23, 33, 35, 37, 41, 42], which were presented in this figure. CPD: cigarettes per day; PY: pack years; the figure for Chang et al. 2006 [23] was estimated from their report that, for paternal preconception smoking, an OR of 1.03 (95% CI: 1.00–1.06) was associated with a one-CPD increment, and an OR of 1.34 (95% CI: 1.02–1.74) with 10-CPD increment, and the figure for Sorahan et al. 1995 [35] was estimated from their report of an OR of 1.16 (95% CI: 1.06–1.27) for change of one level of prenatal use of tobacco products.

Among the 18 studies included in this updated meta-analysis, two studies did not present dose-response analysis [32, 43] and 10 did not find significant dose-response relationships between paternal smoking and childhood ALL. Data from the remaining six studies [23, 33, 35, 37, 41, 42] that previously reported positive dose-response relationships are summarized in Figure 3. These data indicate that dose-response effects may occur before conception, during the prenatal period, or after birth. 

**Figure 4:**
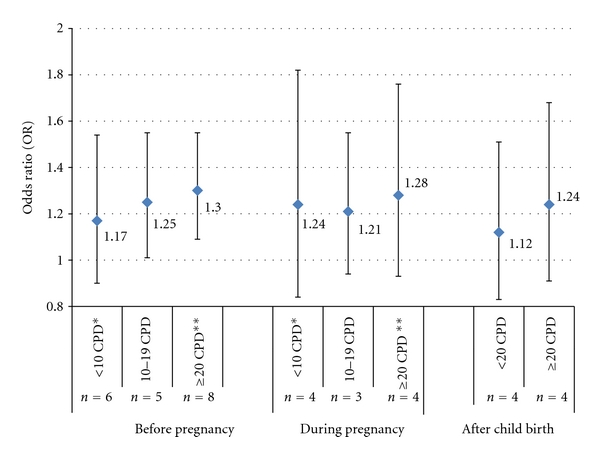
Evaluation of dose-response relationships between paternal smoking at different time windows and childhood ALL risk. Estimates by random-effect models were used when between-study heterogeneity was statistically significant; CPD: cigarettes per day; before pregnancy dose-response analysis was based on eight studies [27–30, 33, 34, 37, 43], during pregnancy analysis was based on four studies [27–29, 33] and after birth analysis was based on four studies [26, 30, 31, 33]; The risk estimates for exposure to paternal smoking with 10–19 CPD and with ≥20 CPD were combined to get estimates on exposure to paternal smoking with <20 CPD from Rudant et al. 2008 [33] and from Menegaux et al. 2005 [31]; *includes exposure to paternal smoking of 1–15 CPD (OR = 0.9, 95% CI: 0.6–1.5) up to child's birth, **includes exposure to paternal smoking of >16 CPD (OR = 0.9, 95% CI: 0.5–1.6) up to child's birth, as reported by Magnani et al. 1990 [29]; The dose-response analysis in Sorahan's paper in 2001 [37] was based on exposure categories of: lifelong nonsmokers, <10, 10–19, 20–29, 30–39, and ≥40 CPD during preconception, the later three categories were combined using the raw data for the purpose of this meta-analysis.

We calculated the summary effects for exposure to different levels of paternal smoking during each of the three time windows, as shown in Figure 4. Data showed a positive dose-response relationship between childhood ALL and preconception paternal smoking, with a summary OR of 1.17 (95% CI: 0.9–1.54), 1.25 (95% CI: 1.01–1.55) and 1.30 (95% CI: 1.09–1.55), for paternal smoking of <10, 10–19, and ≥20 CPD, respectively. For paternal smoking during pregnancy, no dose-response relationship was found. For paternal smoking ≥20 CPD after birth, the summary effect was 1.24 (95% CI: 0.91–1.68), compared to the summary effect of 1.12 (95% CI: 0.83–1.51) for smoking <20 CPD.

## 4. Discussion

### 4.1. Association of Paternal Smoking and Childhood ALL

More than half of the studies included in this analysis reported relative risk estimates that were not statistically significant; one possible reason for this may be that a true association exists, but these studies did not have the sample sizes or statistical power to identify statistically significant associations. This is not surprising given the relatively low summary ORs we identified (i.e., <1.4) and the large sample sizes required to identify ORs of this magnitude in individual studies. Meta-analysis is able to pool all the published data and thus increases study power. The literature review and meta-analysis reported here, which incorporates more studies (*n* = 18) than previous reviews and finds positive dose-response relationships for exposure to paternal smoking before pregnancy and after child birth, supports a stronger association between paternal smoking and childhood ALL. The International Agency for Research on Cancer (IARC) reviewed six studies published up until 2000 in 2004, and their meta-analysis for paternal smoking “*indicated no statistically significant association with acute lymphocytic leukaemia*” [18]. The Surgeon General Report 2006 [20] reviewed 10 epidemiology studies on parental smoking and childhood leukemia published from 1990 to 2001 and concluded that “*The evidence is suggestive but not sufficient to infer a causal relationship between prenatal and postnatal exposure to secondhand smoke and childhood leukemia.*” This report did not examine risk of ALL specifically. The California EPA also updated its review on parental smoking and childhood acute leukemia in its 2005 report [57], which included 13 studies and concluded that “*evidence to date is suggestive of an association between preconceptional paternal smoking and leukemia risk, but not postconceptional ETS (environmental tobacco smoke) exposure.*” These conclusions were all made on overall childhood leukemia including both ALL and AML and did not differentiate the potential effect of paternal smoking on childhood ALL specifically. 

A recent meta-analysis by Lee et al. [42], based on 12 studies, found that the risk of childhood ALL increased with overall paternal smoking (OR = 1.07, 95% CI: 1.00–1.14, *n* = 5) and smoking before pregnancy (OR = 1.17, 95% CI: 1.04–1.30, *n* = 9) but not after birth. This research group did not evaluate risk during pregnancy. In contrast, in our current meta-analysis, overall lifetime paternal smoking and smoking preconception, during pregnancy and after birth, were all positively associated with childhood ALL. Further, positive dose-response relationships were found for exposure to paternal smoking before pregnancy and after child birth. Our findings, and those of Lee, strengthen the association of paternal smoking and childhood ALL overall, particularly preconception, while the effect of SHS during pregnancy and after birth on ALL risk requires further confirmation.

### 4.2. Limited but Possible Confounding and Bias

We assessed the strength of the observed associations in several ways. In general, moderate heterogeneity (*I* ^2^ ≈ 50%) was observed among studies. Selection bias was assessed by comparing summary effects estimated from all studies with those from studies including population-based controls only and was found to be minimal. Most studies analyzed matched cases and controls for gender and age and adjusted for SES and other potential confounders. Comparison of the summary effects of adjusted ORs with those of crude ORs in eight studies suggested that the adjustment of confounding does not impact the data but it is possible that some unmeasured or residual confounders could have contributed to the observed effects. 

Information bias is another potential issue as most of the studies collected data on paternal smoking from the child's mother, who might not be able to provide accurate information on the exposure. However, because the same approach was used for both cases and controls, information bias from this source was probably nondifferential and would likely bias the estimates towards null. Another source of information bias, recall bias, could arise from parents of cases being more likely to recall an exposure than parents of controls. However, smoking is not generally difficult to recall, so that recall bias is less likely than in studies of exposure to other environmental agents.

Both Egger's and Begg's tests indicated the probability of publication bias for paternal smoking before pregnancy, apparently due to inclusion of two studies [37, 41]. Ji et al. 1997 [41] studied the effect of paternal smoking for relatively longer periods or at higher exposures before conception (5 pack years before the conception); also, they obtained the exposure information by independent interviews with subjects' fathers and mothers so that the exposure assessment from this paper might be less biased than that from other studies. Sorahan et al. 2001 [37] examined the effect of paternal smoking with ≥40 CPD before conception, while the other studies generally used ≥20 CPD as their highest exposure group. Thus, the asymmetric funnel plot does not necessarily indicate evidence of publication bias, but may indicate possible heterogeneity or dose-response effects. The fact that many nonsignificant associations were published (56% of studies included in the current review) further indicates that the probability of publication bias is small or limited.

Maternal smoking is another potentially important confounding factor for the association between paternal smoking and childhood ALL. As data on maternal smoking was not adjusted in many of the studies included here, we were unable to estimate the summary effects of paternal smoking with complete adjustment for maternal smoking. However, the two studies in Asia [41, 42] were conducted in regions with very low smoking rates among women; in one study, none of the mothers of cases or controls smoked [41]; in the other study, only 6% of control mothers smoked [42], both reported positive associations (though not all were significant) between paternal smoking during some time windows and at higher levels of exposures and childhood ALL risk (Table 3). Three American studies [21, 23, 27], which examined both paternal and maternal smoking, found no evidence of an increased risk for exposure to only paternal smoking or only maternal smoking. But one study [23] found a significantly increased ALL risk for exposures to both parents' smoking (paternal preconception smoking and maternal smoking after birth). These five studies are detailed in Table 3. Since most of the studies on maternal smoking and childhood ALL found no association, it is unlikely that the results obtained in this meta-analysis were due to confounding effect by maternal smoking. 

The potential effect of smoking in one time period on outcomes associated with another time period, is another confounding factor. Exposure to paternal ever smoking (in lifetime or during any of the three time periods) was positively associated with childhood ALL, but it was weaker than the effect of exposure in specific time windows, indicating a dilution of the effect of paternal smoking in the time window of interest by paternal ever smoking in lifetime. However, it was difficult to fully differentiate the effects of paternal smoking during different time periods in the current study. None of the studies looked at fathers who smoked exclusively during specific exposure windows or adjusted for paternal smoking in all other time periods. The study by Chang et al. 2006 [23] showed that, compared to children of lifetime nonsmoking fathers, the children of ever smoking fathers who did not smoke during the 3-month preconception period had an OR of 1.10 (95% CI: 0.63–1.91) while the children of fathers who smoked during the 3-month preconception period had an increased OR of 1.35 (95% CI: 0.86–2.10). Thus, including preconception paternal smoking in other time windows of interest might bias the risk estimate away from null.

### 4.3. Potential Mechanisms of Action

Different mechanisms likely underlie ALLs arising from exposure to paternal smoking pre- and postconception, and these are currently poorly understood. Active paternal smoking has been shown to deplete plasma and tissue antioxidant and increase oxidative damage to sperm DNA [58]. It has also been reported that mainstream tobacco smoke can cause paternal germ-line DNA mutation among mature male mice and that mutations accumulate in the spermatogonial stem cells with extended exposures [59]. Two published reviews found a suggestive causal relationship between paternal smoking and all childhood cancers, including also brain cancer and nerve system cancer, with significant increased risk of 10% to 20% [15, 60]. These lines of evidence provide biological plausibility that preconception paternal smoking can cause childhood leukemia. However, the elevated point estimate of the association between paternal smoking in the one or three months before conception was not statistically significant. This might be due to the low power of detection because of the small number of studies (*n* = 5) analyzed in this subgroup. Alternatively, this may indicate that the impact on sperm and short lifespan may not be restricted to exposure during this narrow preconception period. Cigarette smoking has been shown to alter gene expression patterns in airway epithelial cells, some irreversibly [61], and to alter microRNA expression profiling in bronchial cells, indicating possible epigenetic effects [62]. It is possible that sperm-producing cells are negatively impacted by persistent changes in gene or miRNA expression as a result of smoking at earlier times than three months before conception. Further studies are necessary to delineate the effects on sperm in well-defined windows of exposure before conception. 

The biological mechanism underlying ALL arising from exposure during pregnancy or after birth could be mediated through changes in the lymphocyte transcriptome and subsequent effects on the immune system, as has been shown for active smoking [63]. A paternal effect *in utero* might be expected to be weaker than a maternal effect. However, among the many studies which have investigated childhood ALL and maternal smoking during pregnancy, only three reported positive associations [27, 36, 39]. If carcinogenesis is not mediated by maternal smoking during pregnancy, it is less likely to be mediated by paternal smoking during this time window. A possible explanation for the positive association found in this meta-analysis is that most fathers who smoked during the pregnancy likely also smoked before the pregnancy or after birth. Paternal smoking status tends to be constant during different time periods [20]. This means that a risk apparently associated with smoking during or after pregnancy may have actually arisen from preconception exposure.

## 5. Conclusion/Impact

Evidence from the current meta-analysis strongly suggests a positive association between paternal smoking and childhood ALL. Given the high prevalence of smoking among males (35% in developed countries and 50% in developing countries [8]), the association with ALL is of great relevance to public health. Future molecular epidemiology studies should be designed with better assessment of paternal smoking during well-defined time windows. Given that smoking cessation is challenging, identifying the most relevant time window and motivating fathers to quit at least during that time window is one potential strategy to reduce the burden of childhood leukemia. Studies should also facilitate investigation of the underlying toxicological mechanisms, such as genotoxic, transcriptomic, or epigenomic effects on sperm or cord blood.

## Figures and Tables

**Table 1:** Description of the 18 original research studies on paternal smoking and childhood ALL included in the meta-analysis.

Study	Cases/controls	Age (years)	Case recruitment	Control selection	Overall*	Before pregnancy	During pregnancy	After birth
Lee et al. 2009, Korea [42]	106/164	0–18	Incident childhood leukemia cases diagnosed in three hospitals in Seoul between 2003 and 2005	Other patients from the three hospitals where cases came from, matched for age and sex	≥400 cigarettes/life time	number of PYs, ≤10 or >10 PYs	smoking at home during pregnancy	number of PYs, ≤10 or >10 PYs
Rudant et al. 2008, France [33]	647/1681	<15	Cases were identified directly by investigators with support of the national cancer registry in France between 2003 and 2004	Population based, with quota match for age and sex		CPDs from the year prior to the child's birth to the interview	CPDs from the year prior to the child's birth to the interview	CPDs from the year prior to the child's birth to the interview
MacArthur et al. 2008, Canada [28]	351/399	0–14	Incidence case from 5 regions in Canada, diagnosed between 1990 and 1994	From health insurance roll, matched for age, sex, and area for each case	ever smoker	CPDs before pregnancy	CPDs in the year prior to the child birth	
Menegaux et al. 2007, France [30]	407/567	<15	Cases derived from the national registry in 14 regions between 1995 and 1998	Population based, frequency match for age, sex, and area		CPDs in the 3 months before pregnancy		CPD from the child's birth to the diagnosis
Chang et al. 2006, USA [23]	228/306	≤15	hospital diagnosed cases between 1995 and 2002, North California Childhood Leukemia Study	Random selection from birth certificates, individual match for age, sex, and maternal race	ever smoker: ≥100 cigs before diagnosis	CPD in the 3 months before pregnancy		
Menegaux et al. 2005, France [31]	240/142	<15	newly diagnosed acute leukemia cases from 1995 to 1999 in four cities in France	Mostly from departments of orthopedic of the same hospital, matched for age range				CPDs from the index birth to interview
Pang et al. 2003, England [32]	1375/6987	<15	National wide population-based cancer cases diagnosed by regional oncology units between 1991–1994 in Scotland and 1992–1994 in England	Randomly selected from Family Health Serves Authorities lists, and matched for sex, date of birth, and geographical area of residence		ever smoked before conception		
Sorahan et al. 2001, England [37]	139/132	<15	Children first diagnosed with leukemia in 3 areas in England in 1980–1983^§^	From General Practitioners list, matched for sex and date of birth		CPDs		
Infante-Rivard et al. 2000, Canada [26]	486/486	0–9	Cases from tertiary care centers for childhood cancers, diagnosed in 1980–1993, Quebec	Population based from family allowance, matched for age, sex, and area				CPDs between birth and date of diagnosis
Brondum et al. 1999, USA [21]	1618/1986	<15	Newly diagnosed with leukemia via clinical trial registries from 1989 to 1993, CCG study	RDD, individually matched on age, race, area code and exchange	smoking amounts during lifetime	ever smoked one month before pregnancy	ever smoked during the three trimesters	ever smoked during nursing period
Schuz et al. 1999, German [43]	686/2588	<15	From a national wide cancer registry (1992–1997) and from cases diagnosed (1980–1994) and lived in vicinity of nuclear installations	randomly selected from complete files of local offices of registration of residents, matched for area, sex, and similar date of birth (within one year)		CPDs in the last 3 months before pregnancy		
Sorahan et al. 1997b, England [38]	573/573	<16	Children who died from leukemia in England, Wales, and Scotland between 1971 to 1976	From birth registers of local authority areas where cases died, matched by sex and date of birth	current status, 6 levels from 0 to 40 CPD			
Sorahan et al. 1997a, England [36]	367/367	<16	Children who died from leukemia in England, Wales, and Scotland between 1953 to 1955	From birth registers of local authority areas where cases died, matched for sex and date of birth	current status, 4 levels from 0 to 20 CPD			
Ji et al. 1997, China [41]	114/114	<15	Newly diagnosed childhood cancer cases from 1985 to 1991 in Shanghai	Population-based controls from household registry, matched for sex, and year of birth		PYs before conception		PYs after birth
Shu et al. 1996, USA, Canada [34]	191/363	≤18 months	infants newly diagnosed matched for leukemia from 1983 to 1988 via clinical trial registries	RDD, individually matched for year of birth, telephone area code, and exchange number		CPDs in the month prior to pregnancy	CPDs during pregnancy	
Sorahan et al. 1995, England [35]	371/371	<16	Children who died from leukemia in England, Wales, and Scotland between 1977 to 1981^§^	From the birth register of the local authority area in which the case child died, matched for sex and date of birth			CPDs during prenatal period, categorized into 6 levels	
John et al. 1991, USA [27]	47/184	0–14	Incident cases aged 0–14 diagnosed in Denver, Colorado from 1976 to 1983	RDD, matched on age, sex, and geographic area.		CPDs during the 12 months prior to birth	CPDs during the 12 months prior to birth	
Magnani et al. 1990, Italy [29]	142/307	6.1/6.6^#^	Pediatric hospital prevalent cases in Turin Italy, diagnosed between 1974 and 1984	Randomly sampled from medical or surgical wards of the same hospitals, no matches		CPDs up to child's birth	CPDs up to child's birth	

RDD: random digit dialing; CPD: cigarettes per day; PY: package year; *: overall status means without specific exposure time period specified; ^§^: There was a small degree of overlap between cases included by Sorahan et al. 2001 [37] and cases included by Sorahan et al. 1995 [35]; ^#^: mean age of cases at diagnosis: 6.1 years, with standard deviation of 3.6 years and mean age of controls: 6.6 years, with standard deviation of 3.5 years.

**Table 2:** Results of meta-analysis of paternal smoking in different time periods and childhood acute lymphoblastic leukemia (ALL) risk.

Paternal smoking^a^	Studies included	*N*	Fixed-effect model^b^		Random-effect model		*I* ^2^ ^c^
			OR (95% CIs)	*P*	OR (95% CIs)	*P*	(%)
Paternal ever smoking	[21, 23, 26–38, 41–43]	18	1.11 (1.05, 1.16)	.000	1.11 (1.05, 1.18)	.000	18
Overall lifetime ever smoking^d^	[21, 23, 28, 36, 38, 42]	6	1.07 (1.01, 1.14)	.027	—	—	0
Preconception	[21, 23, 27–30, 32–34, 37, 41–43]	13	1.16 (1.03, 1.31)	.016	1.25 (1.08, 1.46)	.002	53
With the highest exposure index	[27–30, 33, 34, 37, 41–43]	10	1.37 (1.13, 1.66)	.001	1.38 (1.11, 1.72)	.004	45
Removing the smallest and greatest ORs	[21, 23, 27, 28, 30, 32–34, 41–43]	11	1.16 (1.03, 1.31)	.013	1.25 (1.08, 1.46)	.003	50
Removing the two greatest ORs	[21, 23, 27–30, 32–34, 37, 41–43]	11	1.15 (1.03, 1.28)	.013	1.20 (1.05, 1.36)	.003	41
Removing the highest weight^e^	[21, 23, 27–30, 33, 34, 37, 41–43]	12	1.25 (1.08, 1.45)	.003	1.31 (1.10, 1.56)	.003	49
Removing the OR from Rudant, 2008^f^	[21, 23, 27–30, 32–34, 37, 41–43]	12	1.10 (1.00, 1.22)	.060	1.15 (1.01, 1.31)	.03	26
With well-defined exposure period^g^	[21, 23, 28, 30, 32, 34, 37, 41–43]	10	1.11 (0.99, 1.23)	.069	1.17 (1.02, 1.35)	.026	33
With paternal smoking during 1 or 3 months before pregnancy	[21, 23, 30, 34, 43]	5	1.13 (0.98, 1.29)	.085	—	—	0
With population-based controls	[21, 23, 27, 28, 30, 32–34, 41, 43]	10	1.16 (1.02, 1.31)	.020	1.25 (1.07, 1.45)	.005	54
During pregnancy	[21, 27–29, 33–35, 42]	8	1.19 (1.07,1.32)	.001	1.24 (1.07,1.43)	.004	54
With the highest exposure index	[27–29, 33]	4	1.34 (1.02, 1.77)	.037	1.28 (0.93, 1.76)	.13	65
With adjusted ORs	[21, 27, 28, 33, 34, 42]	6	1.26 (1.04, 1.51)	.017	1.34 (1.07, 1.68)	.010	60
Removing the smallest and greatest ORs	[21, 27, 28, 33–35]	6	1.19 (1.06, 1.33)	.002	1.25 (1.07, 1.45)	.003	58
Removing the highest weight^h^	[21, 27–29, 33, 34, 42]	7	1.23 (1.03,1.47)	.022	1.28 (1.04,1.58)	.022	59
Removing the OR from Rudant, 2008	[21, 27–29, 34, 35, 42]	7	1.15 (1.06, 1.23)	.000	—	—	0
With well-defined exposure period^i^	[21, 34, 35, 42]	4	1.15 (1.07, 1.25)	.002	1.16 (1.03, 1.31)	.015	25
With population-based controls	[21, 27, 28, 33–35]	6	1.19 (1.06, 1.33)	.002	1.25 (1.07, 1.45)	.003	58
After birth	[21, 26, 30, 31, 33, 41, 42]	7	1.20 (0.97, 1.49)	.092	1.24 (0.96, 1.60)	.092	64
With the highest exposure index	[26, 30, 31, 33, 41, 42]	6	1.35 (1.06, 1.72)	.008	1.33 (1.00, 1.78)	.05	57
Removing the smallest and greatest ORs	[26, 30, 31, 33, 41]	5	1.32 (1.03, 1.70)	.027	1.27 (0.95, 1.68)	.10	58
Removing the highest weight^j^	[26, 30, 31, 33, 41, 42]	6	1.35 (1.06, 1.72)	.008	1.33 (1.00, 1.78)	.05	57
Removing the OR from Rudant, 2008	[21, 26, 30, 31, 41, 42]	5	1.05 (0.89, 1.23)	.58	1.06 (0.89, 1.26)	.25	12
With population-based controls	[21, 30, 31, 33, 41]	5	1.23 (0.96, 1.59)	.11	1.25 (0.92, 1.69)	.15	68

^a^When multiple indices of exposure categories were available, the highest was selected to estimate the summary effects; otherwise, the binary category was selected, except for the analysis of the subgroup with highest exposure;

^b^95% confidence interval (CI) and *P* values were estimated by Shore correction when they were wider or greater than the unadjusted estimates by fixed-effect models. The Shore correction incorporates interstudy heterogeneity;

^c^ *I* ^2^ = 100% × (*Q* − *df*)/*Q*, where Q is Cochran's heterogeneity statistic and df the degrees of freedom, with negative values of *I* ^2^ put equal to zero. *I* ^2^ describes the percentage of total variation across studies that is due to heterogeneity rather than chance

^d^Only included studies that reported an association between childhood ALL and paternal overall smoking status during lifetime

^e^Removed the OR from Pang et al. 2003, which accounted for 42% of the weight;

^f^Rudant et al. 2008 reported risk of childhood ALL for paternal smoking one year before the child birth to the time of interview; the same estimated risk was used for calculating the summary effect of childhood ALL for paternal smoking before conception, during pregnancy and after birth.

^g^Only included ORs for paternal smoking before pregnancy, removed the ORs from Rudant et al. 2008, John et al. 1991 and Magnani et al. 1990, which estimated ORs for paternal smoking in the year (12 months) before birth, preconception, and during the prenatal period;

^h^Removed the OR from Sorahan et al. 1995, which accounted for 70% of the weight;

^i^Only included ORs for paternal smoking during pregnancy and excluded ORs for paternal smoking during the year (or 12 months) prior to birth.

^j^Removed the OR from Brondum et al. 1999, which accounted for 35% of the weight.

**Table 3:** Summary of the five studies detangling effect of paternal smoking from maternal smoking on childhood ALL.

Study	Exposure to parental smoking		Number of case/control	OR (95% CI)	Adjustments
	Paternal smoking	Maternal smoking			
Lee et al. 2009, Korea [42]*	Ever smoked cigarettes				Adjusted for age, sex, birth weight, father's education
	Lifetime nonsmokers	Lifetime nonsmokers	22/41	reference	
	ever	lifetime nonsmokers	84/122	1.3 (0.7, 2.4)	
	pack-years before pregnancy				
	0	lifetime nonsmokers	22/41	reference	
	≤10	lifetime nonsmokers	48/60	1.6 (0.8, 3.1)	
	>10	lifetime nonsmokers	28/33	1.6 (0.8, 3.5)	
	Smoking at home during pregnancy				
	Lifetime nonsmokers	lifetime nonsmokers	22/41	reference	
	yes	lifetime nonsmokers	22/22	2.1 (0.9, 4.9)	
			
	pack-years after birth				
	0	lifetime nonsmokers	27/55	reference	
	≤10	lifetime nonsmokers	64/77	1.7 (0.9, 3.1)	
	**>10**	**lifetime** **nonsmokers**	**11/11**	**3.0 (1.0, 8.8)**	

Ji et al. 1997, China [41]	pack-years before pregnancy				Adjusted for birth weight, income, paternal age, education, and alcohol drinking
	0	lifetime nonsmokers	—	reference	
	≤2	lifetime nonsmokers	—	0.8 (0.2–2.5)	
	2 to 5	lifetime nonsmokers	—	1.0 (0.4–2.7)	
	**≥5**	**Lifetime** **nonsmokers**	*—*	**3.8 (1.3–12.3)**	
	Pack-years after pregnancy				
	0	lifetime nonsmokers	—	reference	
	≤2	lifetime nonsmokers	—	1.1 (0.4, 2.8)	
	2 to 5	lifetime nonsmokers	—	1.8 (0.6, 5.2)	
	≥5	lifetime nonsmokers	—	1.8 (0.6, 5.5)	

Chang et al. 2006, USA [23]	No preconception smoking	no postnatal smoking	144/205	reference	household income and maternal smoking during preconception and pregnancy
	No preconception smoking	postnatal smoking	8/27	0.72 (0.22, 2.38)	
	Preconception smoking	no postnatal smoking	36/47	0.88 (0.51, 1.52)	
	**Preconception** **smoking**	**postnatal** **smoking**	**37/23**	**3.94 (1.25, 12.37)**	

Brondum et al. 1999, USA [21]	Never smoked in the home	never smoking in the home	—	reference	Adjusted for household income, mother's and father's race and education
	Never smoked in the home	ever smoked in the home	—	1.10 (0.88, 1.38)	
	Ever smoked in the home	never smoking in the home	—	1.04 (0.86, 1.26)	
	Ever smoked in the home	ever smoked in the home	—	1.09 (0.91, 1.30)	

John et al. 1991, USA [27]	Not during the year prior to birth	not during the 1st trimester	—	reference	Adjusted for father's education
	Not during the year prior to birth	yes, during the 1st trimester	—	1.9 (0.9, 4.1)	
	Yes, during the year prior to birth	not during the 1st trimester	—	1.4 (0.6, 3.1)	
	Yes, during the year prior to birth	yes, during the 1st trimester	—	1.8 (0.8, 4.0)	

*It was reported that small portion of mothers smoked (the smoking rate was 6.1% for controls' mothers; it was not reported for cases' mothers).

## Abbreviations

ALL: Acute lymphoblastic leukemia
CI: Confidence interval
CPD: Cigarettes per day
DALY: Disability adjusted life years
ETS: Environmental tobacco smoke
IARC: International Agency for Research on Cancer
OR: Odds ratio
OSCC: Oxford Survey of Childhood Cancers
PAHs: Polycyclic aromatic hydrocarbons
PY: Pack year
RR: Relative risk
SES: Social economic status
SHS: Secondhand smoke
WHO: World Health Organization.
